# Fibronectin in Immune Responses in Organ Transplant Recipients

**DOI:** 10.1155/2000/98187

**Published:** 2000

**Authors:** Ana J. Coito, Maria De Sousa, Jerzy W. Kupiec-Weglinski

**Affiliations:** 1 The Dumont-UCLA Transplant Center Division of Liver and Pancreas Transplantation Department of Surgery UCLA School of Medicine Los Angeles CA 90095 USA; 2 The Abel Salazar Institute for Biomedical Sciences Porto 4000 Portugal; 3 The Dumont-UCLA Transplant Center Rm. 77-120 CHS 10833 Le Conte Ave Los Angeles CA 90095 USA

**Keywords:** Fibronectin, Transplantation, Acute Rejection, Chronic Rejection, Tolerance

## Abstract

The immune response to an organ allograft involves perpetuation of T cell infiltration and activation. Advances in understanding the mechanisms of T cell activation have placed particular emphasis on the interactions between the T-cell receptor and antigen presenting cells, with little reference to the fact that in vivo activation occurs in the physical context of extracellular matrix proteins (ECM). Indeed, the possibility that ECM proteins may have a determining role in lymphocyte adhesion and tissue localization and function is now becoming more appreciated in view of growing evidence indicating that integrins and other T cell antigens bind ECM components, with some of these components exerting synergistic effects on T- cell activation. This review focuses on the importance of interactions between lymphocytes and fibronectin, a prominent ECM component, for cell migration and function in organ allograft recipients. It explores novel therapeutic approaches based on the assumption that fibronectin represents an active element in the process of T cell activation in the immune cascade triggered by organ transplantation.


*Developmental Immunology*, 2000, Vol. 7(2-4), pp. 239-248
Reprints available directly from the publisher
Photocopying permitted by license only

© 2000 OPA (Overseas Publishers Association)
N.V. Published by license under the
Harwood Academic Publishers imprint,
part of the Gordon and Breach Publishing Group.
Printed in Malaysia

### Fibronectin in Immune Responses in Organ Transplant Recipients

ANA J. COITO[a*], MARIA DE SOUSA[b] and JERZY W. KUPIEC-WEGLINSKI[a]

[a]*The Dumont-UCLA Transplant Center, Division of Liver and Pancreas Transplantation, Department of Surgery, UCLA School of Medicine, Los Angeles, CA 90095, USA and* [b]*The Abel Salazar Institute for Biomedical Sciences, 4000 Porto, Portugal*

The immune response to an organ allograft involves perpetuation of T cell infiltration and activation. Advances in understanding the mechanisms of T cell activation have placed particular emphasis on the interactions between the T-cell receptor and antigen presenting cells, with little reference to the fact that in vivo activation occurs in the physical context of extracellular matrix proteins (ECM). Indeed, the possibility that ECM proteins may have a determining role in lymphocyte adhesion and tissue localization and function is now becoming more appreciated in view of growing evidence indicating that integrins and other T cell antigens bind ECM components, with some of these components exerting synergistic effects on T- cell activation. This review focuses on the importance of interactions between lymphocytes and fibronectin, a prominent ECM component, for cell migration and function in organ allograft recipients. It explores novel therapeutic approaches based on the assumption that fibronectin represents an active element in the process of T cell activation in the immune cascade triggered by organ transplantation.

*Keywords:* Fibronectin, Transplantation, Acute Rejection, Chronic Rejection, Tolerance

Lymphocytes recirculate between blood and lymph until the trigger of signals that stimulate their adhesion to vascular endothelium or extracellular matrix (ECM) proteins (de Sousa et al., 1991; Springer, 1994). To emigrate through vascular endothelium, lymphocytes have to acquire strong adhesion interactions to the vessel wall while resisting to continuous sheer forces (Diamond et al., 1994). The emerging relevance of lymphocyte – ECM interactions to cell adhesion, migration, and positioning within specific tissue microenvironments emphasize the role ECM proteins may play in the functioning of the immune system in both physiological and pathological conditions (de Sousa et al., 1991).

Fibronectin (FN) is likely a key ECM protein involved in these events. FN is a large dimeric glycoprotein (440–500 kDa) composed of a series of independently folding modular domains known as FN repeats I, II, III (Hynes, 1986). These repeating units contain regions or domains which interact with a number of other matrix molecules as well as cells, and mediates functions such as cell migration, matrix assembly, differentiation, and proliferation (Yamada et al., 1992; Hynes, 1986). Structural diversity in FN arises by regulated alternative splicing of a single gene transcript in three segments termed EIIIA, EIIIB, and V in rats, and ED-A, ED-B, and IIICS respectively in humans (Tamkun et al., 1984; Kornbli-

* Corresponding author; Address for proofs: The Dumont-UCLA Transplant Center, Rm. 77–120 CHS, 10833 Le Conte Ave, Los Angeles, CA 90095, USA. Tel: (310) 794–9480; Fax: (310) 267–2358.

htt et al., 1985). The EIIIA (or ED-A) and EIIIB (or ED-B) domains are either included or excluded as intact type III homology repeats. The V (or IIICS) may be excluded, partially included, or fully included. Alternative splicing of the FN gene allows for the generation of multiple isoforms, with 20 isoforms possible in humans (Schwarzbauer, 1991). The importance of FN in cell migration and differentiation is underscored by the observation that mice made null for this ECM protein die during embryonic development (George et al., 1993; Georges-Labouesse et al., 1996). The role of FN in lymphocyte adhesion, migration, and activation has been illustrated in several studies (Shimizu et al., 1990; Hauzenberger et al., 1994; Ybarrondo et al., 1994; Ostergaard et al., 1995; Hunter et al., 1997). Cellular adhesion to FN is mediated by a superfamily of heterodimeric molecules, the integrins (Hynes, 1987). Lymphocytes have been shown to interact with sequences within the type III repeats of FN primarily via two different receptors of the β1 integrin family, the α4β1 and α5β1 (Guan, et al., 1990; Hemler et al., 1995). Thus, α4β1 integrin, interacts with the connecting segment-1 (CS-1), which is located within the V region, and with a recently described segment KLDAPT located in FN-III5 (Moyano et al., 1997). In addition, it was reported that the activated α4β1integrin is also able to interact with the RGD sequence present in the cell adhesion domain (Sanchez-Aparicio et al., 1994). These findings showing the flexibility of the α4β1 integrin in recognizing other domains in the FN molecule, support the important role this ECM protein may play in an array of lymphocyte biological functions. The other main receptor for FN, the α5β1 integrin, known to be present on a subpopulation of resting CD45RA dim memory T cells (Klingemann et al., 1989), interacts via the RGD sequence. Other integrins have also been identified as alternative receptors for FN, such as α4β7 (Postigo et al., 1993), α3β1 (Yamada et al., 1992), and αvβ1 (Vogel, et al., 1990). The adhesive capacity of integrins expressed on circulating lymphocytes is highly regulated. Thus, adhesion of T cells to FN has been shown to be regulated by the engagement of antigen-specific TCR/CD3 complex, as well as CD2, CD7 and CD28 (Shimizu et al., 1992; Shimizu et al., 1995; Chan et al., 1997). The fact that TCR engagement up-regulates adhesion of T lymphocytes to FN, highlights the importance of this protein in host immune responses. Integrin-dependent binding of lymphocytes to FN is also regulated by a number of chemokines, including the C-C chemokines, such as RANTES, monocyte chemotactic protein 1 (MCP-1) and macrophage inhibitory protein 1 (MIP-1) (Weber et al., 1996; Carr et al., 1996; Carr et al., 1994). Modifications in FN expression may also have a regulatory role in the integrin-mediated lymphocyte adhesion. Indeed, there is good evidence that ECM protein binding can modulate integrin expression (LaFlamme et al., 1992; Delcommenne et al., 1995). This review focuses on the significance of FN expression in organ allograft recipients, and explores novel therapeutic approaches based on the assumption that FN is an active participant in the immune cascade leading to allograft rejection.

### FIBRONECTIN IN ORGAN TRANSPLANT RECIPIENTS

Organ allograft represents a useful in vivo experimental system to examine the role of FN in the immune response in which the effector phase is dependent on the migration of alloreactive cells into the foreign tissue (Kupiec-Weglinski et al., 1993; Coito et al., 1994). In our immunohistological and in situ hybridization studies of rat cardiac allografts, a markedly increased expression of FN, mostly vascular, in the early post-transplant period preceded cellular infiltration (Coito et al., 1994; Coito et al., 1997). This initial up-regulation of vascular FN is a common step in both allo- and iso- transplants and may reflect a response to injury or ischemia that occur during the interval of about 45 minutes of cardiac engraftment (Coito et al., 1997). The initiation of vascular FN synthesis in such an early time-point may represent an important signal that triggers lymphocyte recruitment at the graft site. At later time-points after allotransplantation, cellular FN is predominantly expressed in the myocardium contrasting with cardiac isografts in which myocardial FN expression is absent (Fig. 1 A&B) (Coito et al., 1997). In addition, the simultaneous detection of FN and laminin (LN) in cardiac allografts, by laser scanning confocal microscopy, revealed a preferential accumulation of FN in the interstitial areas where infiltrating mononuclear cells (MNC) localize (Fig. 1 C&D) (Coito et al., 1994). Similarly, sensitized lymphocytes after adoptive transfer to test recipients localize in FN-rich areas of both cardiac transplants (Fig 1E) and lymph nodes (Coito et al., 1994). Moreover, treatment of rats with a neutralizing anti-TNF-α serum significantly prolongs cardiac allograft survival, downregulates the local production of FN and reduces intragraft MNC infiltration (Coito et al., 1995). All these findings support a key role of FN to act as an in vivo adhesive factor for lymphocytes to home to specific tissue microenvironments, including an organ transplant. Our combined immunohistological and in situ hybridization analyzes have also demonstrated that the prime sources of FN in rejecting cardiac allografts are macrophages in the myocardium and smooth muscle and endothelial cells in the vessels (Coito et al., 1997; Coito et al., 1995). It remains to be determined how different cell types regulate FN expression in vivo. While TGF-β1 is well known to increase FN expression by fibroblasts, its role in modulating FN expression in other cell types remains unclear (Roberts et al., 1988; Ignotz et al., 1987). Our data suggest a potential role for TNF-α in modulating macrophage appearance, and potentially FN expression, during cardiac allograft rejection (Coito et al., 1995). Other data also indicate that, in coronary arteriopathy after transplantation, endothelial and smooth muscle cells produced increased FN under the regulation of IL-1β and TNF-α (Clausell et al., 1993; Molossi et al., 1995). Moreover, soluble TNF-α receptor reduces the expression of FN and leukocyte infiltration within areas of intimal thickening (Clausell et al., 1994). In cardiac allografts, FN exists in multiple isoforms with a distinct temporal and spatial pattern of expression (Coito et al., 1997). Newly synthesized FN in cardiac grafts includes EIIIA+, EIIIB+ and CS1 variants that are generated by alternative splicing of FN pre-mRNA. The FN variants that include the EIIIA and EIIIB segments are prominent in FN produced during embryogenesis, and their expression in the adult tissues is minimal unless under certain pathological conditions (Vartio et al., 1987; Carnemolla et al., 1989). For example, adult liver synthesizes the plasma form of FN, and this form excludes the EIIIA and EIIIB domains. However, these EIIIA and EIIIB segments are markedly increased during cutaneous wound healing (Brown et al., 1993; French-Constant et al., 1989), neointimal hyperplasia (Knowlton et al., 1992; Mamuya et al., 1992), and glomerular nephritis (Barnes et al., 1995; Nickeleit et al., 1995). Although, very little is known about the function of the EIIIA and EIIIB domains of FN, by comparing the patterns of EIIIA and EIIIB expression in cardiac allografts and isografts, our data support the hypothesis that these FN variants are involved in lymphocyte adhesion, migration, or differentiation in cardiac allografts. Both EIIIA and EIIIB domains are highly expressed in the myocardium of cardiac allografts associated with increased numbers of infiltrating T cells and appearance of infarcts. In contrast, the FN splicing variants are not detected in the myocardium of cardiac isografts, neither are a similar increase in T cells, nor are infarcts. Recently, it has been shown that EIIIA segment mediates adhesion of fibroblastic cells and promotes the differentiation of lipocytes in the liver to become myofibroblasts (Jarnagin et al., 1994). We think that similar processes may be involved in the lymphocyte biological functions in organ allografts. For example, CD8+ T-lymphocytes-mediated lysis of target cells requires the formation of tight conjugates between these cytotoxic T lymphocytes and the targeted cell. While, the cellular receptors for EIIIA and EIIIB domains are not yet fully characterized, the CS-1 domain recognized by the α4β1 integrin, is known to mediate lymphocyte adhesion (Guan et al., 1990; Wayner et al., 1989). The CS-1 subunit of FN is the first alternatively splicing domain found to be increased in cardiac allografts, it is up-regulated as early as at 3 hours post-transplant, and it is preferentially expressed after day 4 (Coito et al., 1997). The presence of specific FN variants may provide a spatial address at which appropriated co-stimulatory signals could initiate or augment critical T cell signaling events. Supporting this assumption, are our recent findings in rat recipients rendered tolerant to MHC-incompatible cardiac allografts by exogenous immunosuppressive therapy. In long-term tolerant recipients, FN is found up-regulated in the vessels in the absence of other vascular adhesion molecules such as VCAM-1 and ICAM-1, highlighting a putative role for FN in the recruitment of leukocytes at the graft site (Coito et al., 1998). Moreover, despite the high number of MNC infiltrating well-functioning grafts, intramyocardial infiltrating macrophages failed to express FN. This contrasted with long-term control recipients undergoing chronic rejection, in which FN expression was readily detectable in the myocardium (Coito et al., 1998). The observation that MNC do accumulate in the myocardium in the absence of cellular FN in tolerant hosts, indicates other role for this ECM protein that is unrelated to cell migration or tissue positioning. Furthermore, by recreating rejection in tolerant hosts, we have pointed out a novel role for FN in the upregulation of IL-2 and IFN-γ expression, indicating that FN by itself may play an active role in cellular activation (Coito et al., unpublished). Our results are consistent with the concept that FN exerts synergistic effects on T cell activation by acting as a co-stimulator for both CD4+ and CD8+ T cells through TCR (Ostergaard et al., 1995; Matsuyama et al., 1989) and cytokine release. For example, the density of immobilized CD3 or TCR mAb required to induce degranulation and tyrosine phosphorylation of cellular proteins by CD8+ T cells is about 10-fold lower in the presence of FN (Ostergaard et al., 1995). Several studies have also shown that adhesion to FN activates tyrosine phosphorilation of several T cell proteins (Ostergaard et al., 1995; Nojima et al., 1992; Ticchioni et al., 1995). The production of IL-2, IFN-γ, and TNF are also stimulated in vitro by interactions between CD4+ cells and FN (Yamada et al., 1991; McCarthy et al., 1997; Brunmark et al., 1997; Hershkoviz et al., 1993). Moreover, it has been reported that the binding of CD4+ cells to ECM proteins, most likely through conformational changes resulting in better presentation to their receptor, may in turn enhance cytokine activity (Crawford et al., 1998; Lortat-Jacob et al., 1991; Lortat-Jacob et al., 1991). Taken together, these findings support the idea that FN is an active participant in the immune cascade leading to graft rejection.

### FIBRONECTIN – MONONUCLEAR CELL INTERACTIONS: POTENTIAL TARGETS FOR THERAPEUTIC INTERVENTION IN TRANSPLANT RECIPIENTS

That MNC-FN disturbed in vivo adhesive interactions play immunopathological role, and may represent important targets for novel therapeutic interventions, has been documented in both autoimmune disease and transplantation animal models. Multiple-organ MNC infiltration occurs in TGF-β1 knockout mice, followed by cachexia, and death (Hines et al., 1994). In addition, cells from TGF-β1 deficient mice exhibit increased adhesion to FN matrices in vitro. Such an increased adhesion in culture has been inhibited following incubation of cells with synthetic FN peptides that interact with integrins (RGD/CS-1) and/or cell surface proteoglycans (C/H-I/II/III/V). Interestingly, daily systemic injections of FN peptide preparations virtually blocked leukocyte tissue sequestration, and the development of autoimmune-like lesions, and retarded the lethal wasting syndrome characteristic for this model. The efficiency of FN peptides has been also documented in the rat model of erosive polyarthritis (Wahl et al., 1994). Not only were FN peptides inhibitory to acute and chronic synovial pathology, but were also found to prevent and reverse local recruitment, and the evolution of arthritis.

Our own data are consistent with the model in which in vivo interactions between the α4β1 integrin receptor and the cell-associated CS1 motif of FN are critical in the acute allograft rejection cascade (Coito et al., 1998). Indeed, treatment of rat recipients of cardiac allografts with a 7 day course of bioactive CS-1 peptides: (i) abrogated acute rejection, and doubled the transplant survival time, (ii) diminished vascular expression of adhesion molecules such as VCAM-1 and ICAM-1, (iii) reduced intragraft infiltration by CD4+ and CD8+ cells, and (iv) decreased allo-Ag activation at the graft site, as evidenced by decreased

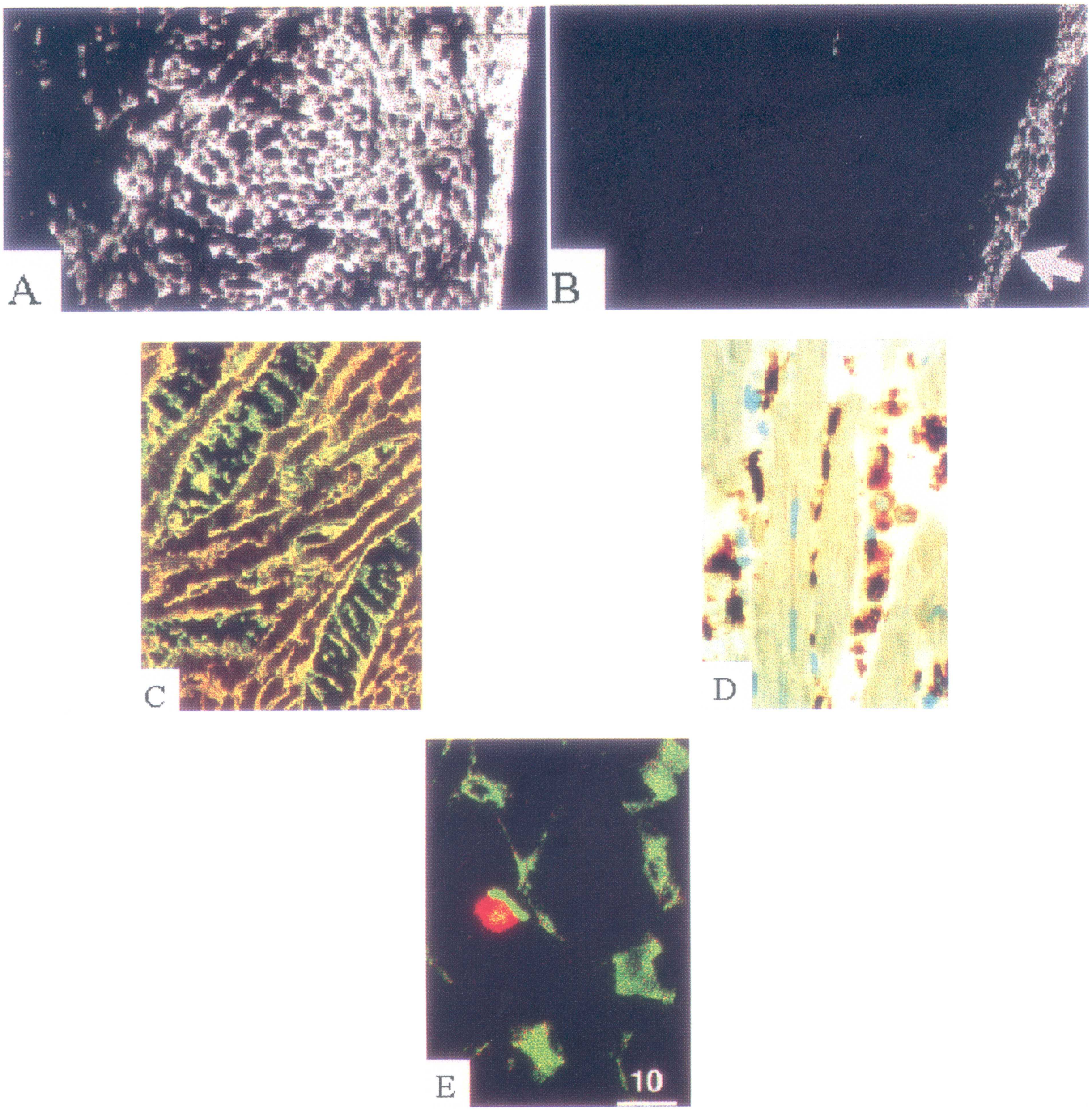

FIGURE 1 FN and infiltrating MNC in cardiac transplants. Immunofluorescence localization of cellular FN is shown in cardiac allograft (A) and isograft (B) at day 6 post-transplant. In cardiac allografts, cellular FN (white) is detected in elevated amounts in both myocardium and epicardium; in cardiac isografts cellular FN is restricted to the epicardium (arrow) but absent from the myocardium. Simultaneous detection of FN and laminin (LN) in rejecting cardiac allograft by confocal microscopy (C) shows a preferential accumulation of FN in the interstitial areas where infiltrating MNC localize (D). The yellow around the myocytes represents mixed deposition of FN and LN; the green in the interstitial areas represents exclusively FN deposits. Panel D shows monocyte/macrophages stained in brown by immunoperoxidase. Panel E illustrates the immunohistologic localization in a cardiac allograft of specifically sensitized lymph node lymphocytes labeled in vitro with Di "I" in red after adoptive transfer. As documented by confocal microscopy, labeled red cells are detected in association with FN deposits (stained in green) at the graft site. Original magnification × 109 (A, B); × 400 (C); × 230 (D); and × 1200 (E) (see Color Plate XIV at the back of this issue)

infiltration by CD25+ cells, and diminished expression of Th1 and Th2 type cytokines. These immunosuppressive effects could be reversed and acute rejection recreated after adjunctive treatment with recombinant IL-2, suggesting that CS1 peptides may induce a temporary state of cytokine-responsive T cell anergy, in vivo.

Most of the adhesion functions mediated by α4β1 integrin are attributed to interactions with its two known ligands, the CS1 motif in the FN molecule, and VCAM-1 expressed on endothelial cell surfaces. Although it is possible the CS1 peptide may interfere with α4β1 – VCAM-1 interactions, α4β1 has been shown to have distinct ligand/binding sites for VCAM-1 and FN (Elices et al., 1990), suggesting that the CS1 peptide that binds to VLA-4 does not likely inhibit the binding by VCAM-1. Moreover, although VCAM-1 and CS1-FN may share spatially overlapping biding sites on α4β1, the concentration of FN peptides that interfere with the α4β1 – VCAM-1 binding are several-fold higher than those required for the α4β1 – CS1 blockade (Makarem et al., 1994). Hence, it seems quite unlikely that the effects of relatively low dose CS1 peptide regimen upon the kinetics of, and local cell recruitment associated with acute graft rejection, could be attributed to the blockade of α4β1 – VCAM-1 interactions. Supporting this notion are findings from mouse cardiac transplant models of persistent T cell infiltration in allografts despite anti-VCAM-1 mAb therapy, and transient T cell infiltrate in isografts. which are devoid of VCAM-1 expression (Orosz et al., 1993). These strongly suggest a role for VCAM-1-independent collateral system(s) that mediate leukocyte sequestration during the course of acute graft-deteriorating rejection. It has been shown that the blockade of α4β1-dependent leukocyte – endothelial adhesive interactions by treatment with anti-VLA-4 mAb (TA-2) was insufficient to delay acute rejection of pancreatic islets in rat recipients (Brown et al., 1993). In contrast to the blockade of α4β1 – FN interactions by CS1 peptides, the protective effects afforded by targeting the α4β1 integrin by mAb alone are rather short-lived, and insignificant upon allo-Ag-driven mononuclear and endothelial cell activation leading to graft rejection in transplant recipients. However, in a more recent study, administration of CS1 peptides to mice recipients of islet allografts not only abrogated graft rejection, but also prevented graft infiltration, suggesting a role for α4/FN interactions in leukocyte homing to the graft site (Stegall et al., 1999).

We have also tested the efficacy of CS-1 peptides in presensitized rat recipients in which therapy with rapamycin (RPM) abrogates rejection at 24h, but it does not prevent the ultimate cardiac allograft loss at 40–50 days (Korom et al., 1998). These long-term grafts show progressive development of arteriosclerosis, a hallmark of chronic rejection (Wasowska et al., 1996). Indeed, an adjunctive course of CS-1 peptides in the early post-transplant phase resulted in the disappearance of the inflammatory and smooth muscle cells from the arterial intimal of long-term recipients. Unlike medium or large arteries in rats undergoing monotherapy, those following adjunctive CS-1 peptides had normal internal elastic laminea, and were free of neointimal thickening, confirming that CS-1 peptides successfully prevented the development of arterial chronic injury. Moreover, treatment with CS1 peptides reduced gene transcript and product levels for T cell- and macrophage- derived cytokines and chemoattractants, which are known to contribute to the development of progressive chronic-type allograft failure. A similar finding was observed in a cholesterol-fed rabbit model in which treatment with CS-1 peptides specifically blocked vascular changes, and markedly diminished accelerated coronary arteriopathy (Molossi et al., 1995). Therapy with FN peptides effectively depressed intragraft transendothelial infiltration by macrophages and T cells, documenting the functional role of FN in the trafficking of inflammatory cells in progression of cardiac arteriopathy. However, unlike in our rat study, CS1 peptides did not affect the grade of cardiac rejection in the rabbit model, as judged by the extensive myocardial cell infiltration, myocyte necrosis, hemorrhage, and fibrosis. As integrin – FN interactions are critical for T cell activation, adhesion, and local retention to perpetuate chronic inflammatory responses, antagonism of cellular activation, and recruitment by FN peptides provides an important mechanism for modulating the multi-step adhesion process, and attenuating aberrant inflammatory responses (Rodriguez et al., 1992). Given the efficacy of FN peptides to block neointimal thickening in coronary vessels, this novel therapy may interfere with the migration of smooth muscle cells from the media into the intima, with resultant reduction of neointimal hyperplasia, consistent with the expression of α4β1 integrin by smooth muscle cells (Molossi et al., 1995). In addition to regulating cell growth and differentiation, FN may also function as a lymphocyte survival factor in cardiac allografts. Indeed, there is a growing body of evidence that anchorage-dependent cells when prevented from attaching to ECM proteins may undergo apoptosis (Meredith et al., 1993; Boudreau et al., 1995; Sethi et al., 1999).

The CS1-mediated prevention of chronic rejection may result from a far more reaching effect on long-term in vivo interactions between MNC, endothelial vascular lining and the ECM scaffolding. Treatment with RPM abrogates rejection in presensitized hosts by depressing humoral and T cell cytotoxic and proliferative responses (Wieder et al., 1993; Schmidbauer et al., 1994). At this point, host-vs-graft interactions are limited to the cellular contact with ECM and the endothelial lining. Adjunctive administration of CS1 peptides during this early post-transplant phase may impair the engagement of T cells with FN as part of the transplant structural framework. At the same time, CS1 peptides may modify intragraft vasculature by reducing its adhesiveness for host leukocytes. During the ensuing maintenance phase, the alterations in intragraft MNC homing and positioning are mimicked by the prevailing macrophage response. The perpetual cycle of late macrophage-associated proinflammatory mediators, smooth muscle proliferation, neointimal formation, and subsequent vasculature occlusion fails to be initiated.

Collectively, these data advance the hypothesis that local synthesis of FN is an ongoing feature of, and adhesive FN – MNC associations are critical for the development of acute and chronic rejection in transplant recipients. Further elucidation of FN – MNC interactions may ultimately offer potential novel sites for intervention in the control of transplant rejection, and may lead to the development of refined strategies based upon new concepts of host immunomodulation.

### Acknowledgements

Authors own work presented in this article has been supported by NIH grants RO1 AI23847 and RO1 AI42223.

### References

Barnes, J.L.; Torres, E.S.; Mitchell, R.J. and Peters, J.H. (1995) Expression of alternatively spliced fibronectin variants during remodeling in proliferative glomerulonephritis. *Am J Pathol* **147**: 1361–1371.

Boudreau, N.; Sympson, C.J.; Werb, Z. and Bissell, M.J. (1995) Suppression of ICE and apoptosis in mammary epithelial cells by extracellular matrix. *Science* **267**: 891–893.

Brown, L.F.; Dubin, D.; Lavigne, L.; Logan, B.; Dvorak, H.F.; and Van de Water, L. (1993) Macrophages and fibroblasts express embryonic fibronectins during cutaneous wound healing. Am J Pathol **142**: 793–801.

Brunmark, A.nd O'Rourke, A.M. (1997) Augmentation of mature CD4+ T cell responses to isolated antigenic class II proteins by fibronectin and intercellular adhesion molecule-1. *J Immunol* **159**: 1676–1685.

Carnemolla, B.; Balza, E.; Siri, A.; Zardi, L.; Nicotra, M.R.; Bigotti, A. and Natali, P.G. (1989) A tumor-associated fibronectin isoform generated by alternative splicing of messenger RNA precursors. *J Cell Biol* **108**: 1139–1148.

Carr, M.W.; Alon, R. and Springer, T.A. (1996) The C-C chemokine MCP-1 differentially modulates the avidity of beta 1 and beta 2 integrins on T lymphocytes. Immunity **4**: 179–187.

Carr, M.W.; Roth, S.J.; Luther, E.; Rose, S.S. and Springer, T.A. (1994) Monocyte chemoattractant protein 1 acts as a T-lymphocyte chemoattractant. *Proc Natl Acad Sci U S A* **91**: 3652–3656.

Chan, A.S.; Mobley, J.L.; Fields, G.B. and Shimizu, Y. (1997) CD7-mediated regulation of integrin adhesiveness on human T cells involves tyrosine phosphorylation-dependent activation of phosphatidylinositol 3-kinase. *J Immunol* **159**: 934–942.

Clausell, N.; Molossi, S. and Rabinovitch, M. (1993) Increased interleukin-1 beta and fibronectin expression are early features of the development of the postcardiac transplant coronary arteriopathy in piglets. *Am J Pathol* **142**: 1772–1786.

Clausell, N.; Molossi, S.; Sett, S. and Rabinovitch, M. (1994) In vivo blockade of tumor necrosis factor-alpha in cholesterol-fed rabbits after cardiac transplant inhibits acute coronary artery neointimal formation. *Circulation* **89**: 2768–2779.

Coito, A.J.; Binder, J.; Brown, L.F.; de Sousa, M.; Van de Water, L. and Kupiec-Weglinski, J.W. (1995) Anti-TNF-alpha treatment down-regulates the expression of fibronectin and decreases cellular infiltration of cardiac allografts in rats. *J Immunol* **154**: 2949–2958.

Coito, A.J.; Binder, J.; de Sousa, M. and Kupiec-Weglinski, J.W. (1994) The expression of extracellular matrix proteins during accelerated rejection of cardiac allografts in sensitized rats. *Transplantation* **57**: 599–605.

Coito, A.J.; Brown, L.F.; Peters, J.H.; Kupiec-Weglinski, J.W. and Van de Water, L. (1997) Expression of fibronectin splicing variants in organ transplantation: a differential pattern between rat cardiac allografts and isografts. *Am J Pathol* **150**: 1757–1772.

Coito, A.J.; de Sousa, M. and Kupiec-Weglinski, J.W. (1994) The role of cellular and extracellular matrix adhesion proteins in organ transplantation. *Cell Adhes Commun* **2**: 249–255.

Coito, A.J.; Korom, S.; Graser, E.; Volk, H.D.; Van de Water, L. and Kupiec-Weglinski, J.W. (1998) Blockade of very late antigen-4 integrin binding to fibronectin in allograft recipients: I. Treatment with connecting segment-1 peptides prevents acute rejection by suppressing intragraft mononuclear cell accumulation, endothelial activation, and cytokine expression. *Transplantation* **65**: 699–706.

Coito, A.J.; Onodera, K.; Kupiec-Weglinski, J.W. (1998) Distinct roles of fibronectin expression by endothelial cells and macrophages in the infectious tolerance pathway in allograft recipients. Surg. Forum, 373–375.

Crawford, S.E.; Stellmach, V.; Murphy-Ullrich, J.E.; Ribeiro, S.M.; Lawler, J.; Hynes, R.O.; Boivin, G.P. and Bouck, N. (1998) Thrombospondin-1 is a major activator of TGF-beta1 in vivo. *Cell* **93**: 1159–1170.

Delcommenne, M. and Streuli, C.H. (1995) Control of integrin expression by extracellular matrix. *J Biol Chem* **270**: 26794–26801.

de Sousa, M.; Tilney, N.L. and Kupiec-Weglinski, J.W. (1991) Recognition of self within self: specific lymphocyte positioning and the extracellular matrix. *Immunol Today* **12**: 262–266.

Diamond, M.S. and Springer, T.A. (1994) The dynamic regulation of integrin adhesiveness. *Curr Biol* **4**: 506–517.

Elices, M.J.; Osborn, L.; Takada, Y.; Crouse, C.; Luhowskyj, S.; Hemler, M.E. and Lobb, R.R. (1990) VCAM-1 on activated endothelium interacts with the leukocyte integrin LVA-4 at a site distinct from the VLA-4/fibronectin binding site. *Cell* **60**: 577–584.

French-Constant, C.; Van de Water, L.; Dvorak, H.F. and Hynes, R.O. (1989) Reappearance of an embryonic pattern of fibronectin splicing during wound healing in the adult rat. *J Cell Biol* **109**: 903–914.

George, E.L.; Georges-Labouesse, E.N.; Patel-King, R.S.; Rayburn, H. and Hynes, R.O. (1993) Defects in mesoderm, neural tube and vascular development in mouse embryos lacking fibronectin. *Development* **119**: 1079–1091.

Georges-Labouesse, E.N.; George, E.L.; Rayburn, H. and Hynes, R.O. (1996) Mesodermal development in mouse embryos mutant for fibronectin. *Dev Dyn* **207**: 145–156.

Guan, J.L. and Hynes, R.O. (1990) Lymphoid cells recognize an alternatively spliced segment of fibronectin via the integrin receptor alpha 4 beta 1. *Cell* **60**: 53–61.

Hauzenberger, D.; Klominek, J. and Sundqvist, K.G. (1994) Functional specialization of fibronectin-binding beta 1-integrins in T lymphocyte migration. *J Immunol* **153**: 960–971.

Hemler, M.E. and Lobb, R.R. (1995) The leukocyte beta 1 integrins. *Curr Opin Hematol* **2**: 61–67.

Hershkoviz, R.; Gilat, D.; Miron, S.; Mekori, Y.A.; Aderka, D.; Wallach, D.; Vlodavsky, I.; Cohen, I.R. and Lider, O. (1993) Extracellular matrix induces tumour necrosis factor-alpha secretion by an interaction between resting rat CD4+ T cells and macrophages. *Immunology* **78**: 50–57.

Hines, K.L.; Kulkarni, A.B.; McCarthy, J.B.; Tian, H.; Ward, J.M.; Christ, M.; McCartney-Francis, N.L.; Furcht, L.T.; Karlsson, S. and Wahl, S.M. (1994) Synthetic fibronectin peptides interrupt inflammatory cell infiltration in transforming growth factor beta 1 knockout mice. *Proc Natl Acad Sci U S A* **91**: 5187–5191.

Hunter, A.J. and Shimizu, Y. (1997) Alpha 4 beta 1 integrin-mediated tyrosine phosphorylation in human T cells: characterization of Crk- and Fyn-associated substrates (pp105, pp115, and human enhancer of filamentation-1) and integrin-dependent activation of p59fyn1. *J Immunol* **159**: 4806–4814.

Hynes, R.O. (1986) Fibronectins. *Sci Am* **254**, 42–51.

Hynes, R.O. (1987) Integrins: a family of cell surface receptors. *Cell* **48**: 549–554.

Hynes, R.O. (1995) Cell adhesion and human disease. Chairman's introduction. *Ciba Found Symp* **189**: 1.

Ignotz, R.A.; Endo, T. and Massague, J. (1987) Regulation of fibronectin and type I collagen mRNA levels by transforming growth factor-beta. *J Biol Chem* **262**: 6443–6446.

Jarnagin, W.R.; Rockey, D.C.; Koteliansky, V.E.; Wang, S.S. and Bissell, D.M. (1994) Expression of variant fibronectins in wound healing: cellular source and biological activity of the EIIIA segment in rat hepatic fibrogenesis. *J Cell Biol* **127**:2037–2048.

Klingemann, H.G. and Dedhar, S. (1989) Distribution of integrins on human peripheral blood mononuclear cells. *Blood* **74**: 1348–1354.

Knowlton, A.A.; Connelly, C.M.; Romo, G.M.; Mamuya, W.; Apstein, C.S. and Brecher, P. (1992) Rapid expression of fibronectin in the rabbit heart after myocardial infarction with and without reperfusion. *J Clin Invest* **89**: 1060–1068.

Kornblihtt, A.R.; Umezawa, K.; Vibe-Pedersen, K. and Baralle, F.E. (1985) Primary structure of human fibronectin: differential splicing may generate at least 10 polypeptides from a single gene. *EMBO. J* **4**: 1755–1759.

Korom, S.; Hancock, W.W.; Coito, A.J. and Kupiec-Weglinski, J.W. (1998) Blockade of very late antigen-4 integrin binding to fibronectin in allograft recipients. II. Treatment with connecting segment-1 peptides prevents chronic rejection by attenuating arteriosclerotic development and suppressing intragraft T cell and macrophage activation. *Transplantation* **65**: 854–859.

Kupiec-Weglinski, J.W.; Heemann, U.W.; Coito, A.J.; Tullius, S.G.; Tilney, N.L. and de Sousa, M. (1993) Adhesion molecule interaction with extracellular matrix. *Exp Nephrol* **1**: 78–82.

LaFlamme, S.E.; Akiyama, S.K. and Yamada, K.M. (1992) Regulation of fibronectin receptor distribution [published erratum appears in J Cell Biol 1992 Jul; 118(2):491]. *J Cell Biol* **117**: 437–447.

Lortat-Jacob, H. and Grimaud, J.A. (1991) Interferon-gamma C-terminal function: new working hypothesis. Heparan sulfate and heparin, new targets for IFN-gamma, protect, relax the cytokine and regulate its activity. *Cell Mol Biol* **37**: 253–260.

Lortat-Jacob, H.; Kleinman, H.K. and Grimaud, J.A. (1991) High-affinity binding of interferon-gamma to a basement membrane complex (matrigel). *J Clin Invest* **87**: 878–883.

Makarem, R.; Newham, P.; Askari, J.A.; Green, L.J.; Clements, J.; Edwards, M.; Humphries, M.J. and Mould, A.P. (1994) Competitive binding of vascular cell adhesion molecule-1 and the Hep II/IIICS domain of fibronectin to the integrin alpha 4 beta 1. *J Biol Chem* **269**: 4005–4011.

Mamuya, W.S. and Brecher, P. (1992) Fibronectin expression in the normal and hypertrophic rat heart. *J Clin Invest* **89**: 392–401.

Matsuyama, T.; Yamada, A.; Kay, J.; Yamada, K.M.; Akiyama, S.K.; Schlossman, S.F. and Morimoto, C. (1989) Activation of CD4 cells by fibronectin and anti-CD3 antibody. A synergistic effect mediated by the VLA-5 fibronectin receptor complex. *J Exp Med* **170**: 1133–1148.

McCarthy, J.B.; Vachhani, B.V.; Wahl, S.M.; Finbloom, D.S. and Feldman, G.M. (1997) Human monocyte binding to fibronectin enhances IFN-gamma-induced early signaling events. *J Immunol* **159**: 2424–2430.

Meredith, J.E.J.; Fazeli, B. and Schwartz, M.A. (1993) The extracellular matrix as a cell survival factor. *Mol Biol Cell* **4**,: 953–961.

Molossi, S.; Clausell, N. and Rabinovitch, M. (1995) Reciprocal induction of tumor necrosis factor-alpha and interleukin-1 beta activity mediates fibronectin synthesis in coronary artery smooth muscle cells. *J Cell Physiol* **163**: 19–29.

Molossi, S.; Elices, M.; Arrhenius, T.; Diaz, R.; Coulber, C. and Rabinovitch, M. (1995) Blockade of very late antigen-4 integrin binding to fibronectin with connecting segment-1 peptide reduces accelerated coronary arteriopathy in rabbit cardiac allografts [see comments]. *J Clin Invest* **95**: 2601–2610.

Moyano, J.V.; Carnemolla, B.; Dominguez-Jimenez, C.; Garcia-Gila, M.; Albar, J.P.; Sanchez-Aparicio, P.; Leprini, A.; Querze, G.; Zardi, L. and Garcia-Pardo, A. (1997) Fibronectin type III5 repeat contains a novel cell adhesion sequence, KLDAPT, which binds activated alpha4beta1 and alpha4beta7 integrins. *J Biol Chem* **272**: 24832–24836.

Nickeleit, V.; Zagachin, L.; Nishikawa, K.; Peters, J.H.; Hynes, R.O. and Colvin, R.B. (1995) Embryonic fibronectin isoforms are synthesized in crescents in experimental autoimmune glomerulonephritis. *Am J Pathol* **147**: 965–978.

Nojima, Y.; Rothstein, D.M.; Sugita, K.; Schlossman, S.F. and Morimoto, C. (1992) Ligation of VLA-4 on T cells stimulates tyrosine phosphorylation of a 105-kD protein. *J Exp Med* **175**: 1045–1053.

Orosz, C.G.; Ohye, R.G.; Pelletier, R.P.; Van Buskirk, A.M.; Huang, E.; Morgan, C.; Kincade, P.W. and Ferguson, R.M. (1993) Treatment with anti-vascular cell adhesion molecule 1 monoclonal antibody induces long-term murine cardiac allograft acceptance. *Transplantation* **56**: 453–460.

Ostergaard, H.L. and Ma, E.A. (1995) Fibronectin induces phosphorylation of a 120-kDa protein and synergizes with the T cell receptor to activate cytotoxic T cell clones. *Eur J Immunol* **25**, 252–256.

Postigo, A.A.; Sanchez-Mateos, P.; Lazarovits, A.I.; Sanchez-Madrid, F. and de Landazuri, M.O. (1993) Alpha 4 beta 7 integrin mediates B cell binding to fibronectin and vascular cell adhesion molecule-1. Expression and function of alpha 4 integrins on human B lymphocytes. *J Immunol* **151**: 2471–2483.

Roberts, C.J.; Birkenmeier, T.M.; McQuillan, J.J.; Akiyama, S.K.; Yamada, S.S.; Chen, W.T.; Yamada, K.M. and McDonald, J.A. (1988) Transforming growth factor beta stimulates the expression of fibronectin.

and of both subunits of the human fibronectin receptor by cultured human lung fibroblasts. *J Biol Chem* **263**: 4586–4592.

Rodriguez, R.M.; Pitzalis, C.; Kingsley, G.H.; Henderson, E.; Humphries, M.J. and Panayi, G.S. (1992) T lymphocyte adhesion to fibronectin (FN): a possible mechanism for T cell accumulation in the rheumatoid joint. *Clin Exp Immunol* **89**: 439–445.

Sanchez-Aparicio, P.; Dominguez-Jimenez, C. and Garcia-Pardo, A. (1994) Activation of the alpha 4 beta 1 integrin through the beta 1 subunit induces recognition of the RGDS sequence in fibronectin. *J Cell Biol* **126**: 271–279.

Schmidbauer, G.; Hancock, W.W.; Wasowska, B.; Badger, A.M. and Kupiec-Weglinski, J.W. (1994) Abrogation by rapamycin of accelerated rejection in sensitized rats by inhibition of alloantibody responses and selective suppression of intragraft mononuclear and endothelial cell activation, cytokine production, and cell adhesion. *Transplantation* **57**: 933–941.

Schwarzbauer, J.E. (1991) Fibronectin: from gene to protein. *Curr Opin Cell Biol* **3**: 786–791.

Sethi, T.; Rintoul, R.C.; Moore, S.M.; MacKinnon, A.C.; Salter, D.; Choo, C.; Chilvers, E.R.; Dransfield, I.; Donnelly, S.C.; Strieter, R. and Haslett, C. (1999) Extracellular matrix proteins protect small cell lung cancer cells against apoptosis: a mechanism for small cell lung cancer growth and drug resistance in vivo. *Nat Med* **5**: 662–668.

Shimizu, Y.; Mobley, J.L.; Finkelstein, L.D. and Chan, A.S. (1995) A role for phosphatidylinositol 3-kinase in the regulation of beta 1 integrin activity by the CD2 antigen. *J Cell Biol* **131**: 1867–1880.

Shimizu, Y.; van Seventer, G.A.; Ennis, E.; Newman, W.; Horgan, K.J. and Shaw, S. (1992) Crosslinking of the T cell-specific accessory molecules CD7 and CD28 modulates T cell adhesion. *J Exp Med* **175**: 577–582.

Shimizu, Y.; van Seventer, G.A.; Horgan, K.J. and Shaw, S. (1990) Costimulation of proliferative responses of resting CD4+ T cells by the interaction of VLA-4 and VLA-5 with fibronectin or VLA-6 with laminin. *J Immunol* **145**: 59–67.

Springer, T.A. (1994) Traffic signals for lymphocyte recirculation and leukocyte emigration: the multistep paradigm. *Cell* **76**: 301–314.

Stegall, M.D.; Elices, M.; Pietra, W.; Shepard, G.; Gup, C.; Gill, R.G. (1999). Different roles for alpha 4-integrin/VCAM-1 and alpha 4/fibronectin interactions in allograft rejection. Transplant Proc 31: 786.

Tamkun, J.W.; Schwarzbauer, J.E. and Hynes, R.O. (1984) A single rat fibronectin gene generates three different mRNAs by alternative splicing of a complex exon. *Proc Natl Acad Sci U S A* **81**: 5140–5144.

Ticchioni, M.; Deckert, M.; Bernard, G.; Calandra, D.; Breittmeyer, J.P.; Imbert, V.; Peyron, J.F. and Bernard, A. (1995) Comitogenic effects of very late activation antigens on CD3-stimulated human thymocytes. Involvement of various tyrosine kinase pathways. *J Immunol* **154**: 1207–1215.

Vartio, T.; Laitinen, L.; Narvanen, O.; Cutolo, M.; Thornell, L.E.; Zardi, L. and Virtanen, I. (1987) Differential expression of the ED sequence-containing form of cellular fibronectin in embryonic and adult human tissues. *J Cell Sci* **88 (Pt 4)**: 419–430.

Vogel, B.E.; Tarone, G.; Giancotti, F.G.; Gailit, J. and Ruoslahti, E. (1990) A novel fibronectin receptor with an unexpected subunit composition (alpha V beta 1). *J Biol Chem* **265**, 5934–5937.

Wahl, S.M.; Allen, J.B.; Hines, K.L.; Imamichi, T.; Wahl, A.M.; Furcht, L.T. and McCarthy, J.B. (1994) Synthetic fibronectin peptides suppress arthritis in rats by interrupting leukocyte adhesion and recruitment. *J Clin Invest* **94**: 655–662.

Wasowska, B.; Wieder, K.J.; Hancock, W.W.; Zheng, X.X.; Berse, B.; Binder, J.; Strom, T.B. and Kupiec-Weglinski, J.W. (1996) Cytokine and alloantibody networks in long term cardiac allografts in rat recipients treated with rapamycin. *J Immunol* **156**: 395–404.

Wayner, E.A.; Garcia-Pardo, A.; Humphries, M.J.; McDonald, J.A. and Carter, W.G. (1989) Identification and characterization of the T lymphocyte adhesion receptor for an alternative cell attachment domain (CS-1) in plasma fibronectin. *J Cell Biol* **109**: 1321–1330.

Weber, C.; Alon, R.; Moser, B. and Springer, T.A. (1996) Sequential regulation of alpha 4 beta 1 and alpha 5 beta 1 integrin avidity by CC chemokines in monocytes: implications for transendothelial chemotaxis. *J Cell Biol* **134**: 1063–1073.

Wieder, K.J.; Hancock, W.W.; Schmidbauer, G.; Corpier, C.L.; Wieder, I.; Kobzik, L.; Strom, T.B. and Kupiec-Weglinski, J.W. (1993) Rapamycin treatment depresses intragraft expression of KC/MIP-2, granzyme B, and IFN-gamma in rat recipients of cardiac allografts. *J Immunol* **151**: 1158–1166.

Yamada, A.; Nikaido, T.; Nojima, Y.; Schlossman, S.F. and Morimoto, C. (1991) Activation of human CD4 T lymphocytes. Interaction of fibronectin with VLA-5 receptor on CD4 cells induces the AP-1 transcription factor. *J Immunol* **146**: 53–56.

Yamada, K.M.; Aota, S.; Akiyama, S.K. and LaFlamme, S.E. (1992) Mechanisms of fibronectin and integrin function during cell adhesion and migration. *Cold Spring Harb Symp Quant Biol* **57**: 203–212.

Ybarrondo, B.; O'Rourke, A.M.; Brian, A.A. and Mescher, M.F. (1994) Contribution of lymphocyte function-associated-1/intercellular adhesion molecule-1 binding to the adhesion/signaling cascade of cytotoxic T lymphocyte activation. *J Exp Med* **179**: 359–363.

